# A Comparative Study on Cyclodextrin Derivatives in Improving Oral Bioavailability of Etoricoxib as a Model Drug: Formulation and Evaluation of Solid Dispersion-Based Fast-Dissolving Tablets

**DOI:** 10.3390/biomedicines11092440

**Published:** 2023-09-01

**Authors:** Doaa Elsegaie, Mohamed A. El-Nabarawi, Hanaa Abdelmonem Mahmoud, Mahmoud Teaima, Dina Louis

**Affiliations:** 1 Department of Pharmaceutics and Industrial Pharmacy, Faculty of Pharmacy, Heliopolis University, Cairo 11785, Egypt; doaa.mohamed@hu.edu.eg; 2 Department of Pharmaceutics and Industrial Pharmacy, Faculty of Pharmacy, Cairo University, Cairo 11562, Egypt; mohamed.elnabarawi@pharma.cu.edu.eg (M.A.E.-N.); hanaa.elsaghir@pharma.cu.edu.eg (H.A.M.); mahmoud.teaima@pharma.cu.edu.eg (M.T.)

**Keywords:** etoricoxib, solid dispersion, factorial design, carrier, Prosolv ODT^®^, F-melt type C^®^

## Abstract

Etoricoxib, as a model drug, has a poor solubility and dissolution rate. Cyclodextrin derivatives can be used to solve such a problem. A comparative study was run on three cyclodextrin derivatives, namely β-CD, HP β-CD, and SBE β-CD, to solve the drug problem through the formulation of solid dispersions and their preparation into fast-dissolving tablets. Preparations utilized different (1:1, 1:2, and 1:4) drug:carrier ratios. Nine fast-dissolving tablets (containing 1:4 drug: carrier) were formulated using Prosolv ODT^®^ and/or F-melt^®^ type C as super-disintegrants. Optimized formulation was chosen based on a 3^2^ factorial design. The responses chosen were the outcomes of the in vitro evaluation tests. The optimized formulation that had the highest desirability (0.86) was found to be SD-HP3, which was prepared from etoricoxib: HP β-CD at a 1:4 ratio using equal amounts of Prosolv ODT^®^ and F-melt^®^ type C. An in vivo evaluation of SD-HP3 on a rabbit model revealed its superiority over the marketed product Arcoxia^®^. SD-HP3 showed a significantly lower Tmax (13.3 min) and a significantly higher Cmax (9122.156 μg/mL), as well as a significantly higher AUC, than Arcoxia^®^. Thus, the solubility, dissolution, and bioavailability of etoricoxib were significantly enhanced.

## 1. Introduction

Poorly aqueous soluble drugs have low bioavailability due to impaired absorption, which is a major concern for pharmaceutical companies worldwide [1]. Several methods were used to increase their solubility. Among these was the solid dispersion technique, which has provoked the interest of researchers as an efficient method of increasing the dissolution rate, resulting in increased bioavailability of a variety of weakly water-soluble medicines [2]. Owing to increased wettability, improved dispersibility of drug particles, the presence of the drug in amorphous form, and the absence of drug-particle aggregation due to the use of various carriers, fast and quick drug dissolution from solid dispersions has been observed [3,4].

Etoricoxib, the 5-chloro-6’-methyl-3-[4-(methylsulfonyl) phenyl]-2,3-bipyridine, is an oral analgesic and non-steroidal anti-inflammatory drug (NSAID) that is a highly selective second-generation cyclooxygenase-2 (COX-2) inhibitor [5,6]. It has shown some improvement in efficacy compared to common NSAIDs [7]. It is used for the treatment of rheumatoid arthritis, osteoarthritis, postsurgical dental pain, chronic back pain, and acute gout [8]. Furthermore, new research has shown that it is effective in patients with ankylosing spondylitis [9]. However, its limited aqueous solubility and poor dissolution can cause formulation challenges [10] and limit its therapeutic use by delaying its absorption and onset of action [11,12].

Cyclodextrins (CDs) have functioned to improve the dissolution properties of drugs through drug encapsulation [13] within their toroidal structure [14]. They have a hydrophilic external face and an inner surface with a certain hydrophobicity. They are the most important organic compounds capable of forming complexes with poorly soluble drugs through non-covalent bonding, such as van der Waals interactions, hydrophobic effects, solvent re-organization, and hydrogen bonding [15,16]. As a result, the host–guest systems are more soluble and stable. Cyclodextrins are commonly used as solubility enhancers in drug formulations due to their ability to form water-soluble inclusion complexes with poorly water-soluble drugs [17]. The cyclodextrin drug conjugate has the benefit of being able to survive passage through the stomach and small intestine, and drug release is initiated through cyclodextrin enzymatic degradation in the colon [17,18,19]. The most commonly used CD derivatives are hydroxypropylated and methylated CDs. Their superior solubilizing, amorphizing, and complexing abilities have been extensively established [20]. Several methods were adopted in the preparation of CD inclusion complexes, namely co-precipitation, kneading, supercritical carbon dioxide, microwave irradiation, the grinding method, and spray-drying [21].

Over the last decade, fast-dissolving tablets have risen in popularity, particularly among the elderly and youngsters who have difficulty swallowing tablets or capsules. Fast-dissolving tablets can break down or dissolve quickly in the oral cavity with saliva, between 15 and 60 s, without the need for water. The disintegrants used should meet this criterion by dissolving the tablets within the time given. For the manufacturing of fast-dissolving tablets, many procedures such as freeze drying, sublimation, spray drying, molding, and direct-compression methods have been documented.

In the present study, a new approach to using cyclodetrins in improving drug-dissolution properties has been adopted. Cyclodextrins have been introduced as carriers rather than complexing agents. The combination of the drug and cyclodextrins was weight-based. This approach was suggested to make use of the cyclodextrins’ advantages without increasing the costs of formulation that can be caused by using large quantities of the cyclodextrins.

The solvent-evaporation approach was used to create solid etoricoxib dispersions using different carriers such as β-CD, HP β-CD, and SBE β-CD to increase its solubility and dissolution rate. The successful solid dispersions were formulated as fast-dissolving tablets and evaluated for bioavailability.

## 2. Materials and Methods

### 2.1. Materials

A sample of etoricoxib was provided by Bright life Pharmaceuticals, Egypt, β-CD (water ≤14%), HP β-CD (DS of 4.9), SBE β-CD (water at least 4%, average DS of 6.5) (Sigma Aldrich—Burlington, MA, USA), F-melt^®^ Type C (a gift sample from FUJI Chemical Industry Co., Ltd., Tokyo, Japan) Prosolv^®^ ODT G2 (a gift sample from JRS Pharma, Rosenberg, Germany), and Ethanol 99.9% (Lab Chem, Cairo, Egypt). All the reagents used were of pharmaceutical grade.

### 2.2. Preparation of Etoricoxib Solid Dispersions and Their Physical Mixtures Using Cyclodextrin Derivatives

Batches of 5 g from each of the physical mixtures or solid dispersions of Etoricoxib with β-CD, HP β-CD, and SBE β-CD were prepared. Physical mixtures were prepared by mixing etoricoxib with β-CD, HP β-CD, and SBE β-CD at 1:1, 1:2, and 1:4, drug: carrier weight ratios in a glass mortar. The mixtures were mixed together for 10 min.

For the preparation of etoricoxib solid dispersion, the solvent evaporation technique was used to create solid dispersions from several cyclodextrin derivatives like β-CD, HP β-CD, and SBE β-CD as carriers at the same drug: carrier ratios in physical mixtures. The cyclodextrins were dispersed in the ethanolic solution as fine particles, and the solvent was removed through evaporation over a 60 °C water bath. [22]. Dry solid dispersions were stored in a desiccator until they attained a consistent mass. Afterwards, they were crushed and passed through standard sieve number 22 (corresponding to 850 μm).

### 2.3. Characterization of Etoricoxib Solid Dispersion and Their Physical Mixtures Using Cyclodextrin Derivatives

#### 2.3.1. Determination of Percentage Yield

The mass of produced solid dispersions was determined, and the percentage yield was calculated based on Equation (1) [23]:

(1) $$Percentage\;yield=\frac{Weight\;of\;prepared\;solid\;dispersion}{Total\;weight\;of\;drug\;and\;carriers}\times 100$$

#### 2.3.2. Determination of Percent Drug Content

To ensure the uniformity of drug content, an accurately weighed amount of 25 mg of solid dispersions of etoricoxib was placed in a 25 mL volumetric flask. Ten milliliters of ethanol were added and thoroughly stirred for one hour with a rotating shaker (V-Tech, Delhi, India). With ethanol, the volume was brought up to the mark. The solution was appropriately diluted with ethanol, the drug absorption was spectrophotometrically recorded (UV-Visible spectrophotometer, Shimadzu, Kyoto, Japan) at λ-max 233 nm, and the drug concentration (practical drug content) was calculated based on the constructed calibration curve of the drug in ethanol. Equation (2) was used to compute the % drug content [3].

(2) $$\mathrm{Percent}\;\mathrm{Drug}\;\mathrm{Content}=\frac{Practical\;drug\;content\;in\;solid\;dispersions}{Theoretical\;drug\;content\;in\;solid\;dispersions}\times 100$$

#### 2.3.3. Determination of Saturation Solubility

For 48 h, a known excess of the samples (etoricoxib solid dispersions, physical mixtures, and pure etoricoxib) containing 10 mg of etoricoxib was mixed with 10 mL of phosphate buffer (pH = 6.8) and 0.1 N HCL (pH = 1.2) and placed in a constant-temperature rotating water bath (Gyromax—Frederick, MD, USA) set to 50 rpm (37 ± 0.5 °C). The samples were then filtered, diluted, and analyzed at 233 nm using a UV-VIS spectrophotometer (Shimadzu). The concentration of etoricoxib was then estimated. The experiment was carried out three times.

#### 2.3.4. In vitro Dissolution Studies

In vitro dissolution studies were performed in 900 mL of phosphate buffer (pH 6.8) and 0.1 N HCL pH (1.2) at 37 ± 0.5 °C and 50 rpm rotation speed using a USP II apparatus (Electrolab, Mumbai, India). The test was carried out on mixtures containing 60 mg of etoricoxib (pure etoricoxib, physical mixes of etoricoxib, and solid dispersions of etoricoxib). Samples (5 mL) were extracted at predetermined intervals and compensated for an equivalent volume of fresh dissolution medium. The withdrawn samples were filtered (45 μm micro-filter), and drug concentration was spectrophotometrically assayed at 233 nm using a UV-VIS spectrophotometer (Shimadzu, Japan) [24].

#### 2.3.5. Fourier Transform-Infrared (FTIR) Spectroscopy

Samples were ground into powder and assessed as KBr pellets using an FTIR (Shimadzu, Japan). An ATR sampling technique was used to examine samples from the drug alone, carrier alone, and solid dispersions of drug and carrier, and the spectrum was scanned over the frequency range between 4000 and 600 cm^−1^ using 20 scans at a 4 cm^−1^ resolution to determine possible interactions between pure drug and carriers.

#### 2.3.6. Differential Scanning Calorimetry (DSC)

Samples (etoricoxib, and solid dispersions) weighing precisely 5–7 mg each were placed into solid aluminum pans with no sealing. A differential scanning calorimeter (Shimadzu Model DT-60) was used to scan the samples from 0 to 300 °C at a constant speed of 10 °C min^−1^.

### 2.4. Pre-Compression Studies of Etoricoxib Solid Dispersion

#### 2.4.1. Preparation of Powder Blend

The successful etoricoxib solid dispersions were mixed via trituration with additives, namely, F melt^®^ type C, and Prosolv^®^ ODT, to formulate fast-dissolving, directly compressible tablets. The directly compressible tablet formulations were designed using a 3^2^ factorial design using the Design Expert^®^ program Version 11. Two factors were involved, namely, the type of cyclodextrin (at three levels: β-CD, HP β-CD and SBE β-CD) and the type of directly compressible vehicle (at three levels: F melt^®^ type C, Prosolv^®^ ODT, and a mixture of equal amounts of both). The formulation codes are given in Table 1. The composition of the 9 suggested formulations is listed in Table 2.

#### 2.4.2. Characterization of the Powder Blend

The prepared powder blends were characterized by measuring several parameters such as powder bulk density [25], tapped density [26], angle of repose [26,27], Carr’s index [28], and Hausner ratio [29].

### 2.5. Preparation of Etoricoxib Tablets by Direct Compression

The powder blends suggested for the 9 formulations to manufacture tablets were compressed using a single-punch tablet machine to produce batches from each formulation consisting of 100 tablets.

### 2.6. Evaluation of Fast-Dissolving Tablets

The prepared tablets were evaluated through the determination of weight variation [30], hardness (fc) using a hardness tester (Monsanto, Düsseldorf, Germany) [31], and tablet friability(F) using a Roche Friabilator (Roche, Basel, Switzerland) [32]. The organoleptic properties of prepared tablets were also investigated by six volunteers to record the color, odor, and taste of tablets [33]. This test was important to measure the power of the formulation to mask the drug taste. All evaluation tests were run as triplicates.

#### 2.6.1. In Vitro Disintegration Time

The in vitro disintegration time was determined using the disintegration test apparatus (Pharma test—Hainburg, Germany). A tablet was placed in each of the six tubes of the apparatus, and one tablet was added to each tube. The time it took for the tablet to completely disintegrate with no edible bulk remaining in the instrument was measured in seconds. [34]

#### 2.6.2. In Vitro Drug Dissolution

The drug’s in vitro dissolution profile was investigated using the USP 2 Paddle apparatus (USP Type II, Labindia Model 2000 Dissoo Tablet Dissolution Tester, Noida, India). The apparatus was run at 50 rpm, at 37 ± 0.5 °C, using 0.1 N HCl (900 mL) and pH 6.8 phosphate buffer (900 mL) as dissolution media, to simulate the dissolution process in the mouth and stomach, respectively. The amount of drug dissolved from tablets was determined spectrophotometrically at λ max of 233 nm [35]. The formulations were compared based on the percentage of drug dissolved after 1 and 10 min in each of the dissolution media. The dissolution efficiency was also calculated.

#### 2.6.3. Optimization of Fast-Dissolving Etoricoxib Tablet

The preceding test findings were used as responses (dependent variables) in the factorial design to evaluate the desirability of formulations and select the optimized formulation for further investigation. Table 3 summarizes the responses together with their constraints. For the in vivo study, the formulation with the highest desirability was chosen.

### 2.7. In vivo Bioavailability Study

The formulation with the highest desirability was selected for the in vivo study and was compared to the marketed formulation, Arcoxia^®^. Twelve white female New Zealand rabbits (weighing 1.5–2 kg) were haphazardly separated into 2 groups with a parallel design. The Cairo University ethical committee approved the study protocol to use animals in research (PI 2962). The rabbits had a 12 h fast while having free access to water. Without anesthesia, a catheter was placed into the anterior vena cava via the marginal ear vein. Group A of rabbits orally received “SD-HP3” containing the equivalent of 30 mg etoricoxib, whereas group B received Arcoxia^®^ at the same equivalent dose as the selected formulation. Blood samples (2 mL) were collected into heparinized tubes at 0, 5, 10, 20, 30, 40, 60, 120, and 240 min after the administration of the treatment. Following the collection of the final blood sample 4 h after dosage, ear catheters were withdrawn, and rabbits were returned to their cages with free access to food and water and supervision if they need additional care.

Centrifugation at 4000 rpm for 20 min separated the plasma from the heparinized whole blood [36,37]. After centrifugation, plasma samples were immediately transferred to Eppendorf tubes and kept at −20 °C until analysis. LC-MS chromatographic analysis was used to determine the drug plasma concentration. At a flow rate of 400 L/min, the analyte was eluted using a mobile phase consisting of 0.05% formic acid and methanol (2:3, *v*/*v*) at a volume of 1 L. The temperature of the auto-sampler was 25 °C. Methanol was utilized as an organic solvent in the mobile phase because it was five times more sensitive to etoricoxib than acetonitrile.

### 2.8. Statistical Analysis of Results

All experiments were run in triplicates. The results of those experiments were analyzed using a one-way ANOVA test, and the results were compared at *p* < 0.05 using SPSS software (IBM SPSS Statistics 22, 2021).

## 3. Results

### 3.1. Percentage Yield and Percent Drug Content

The percentage yield ranged from 82.5% ± 0.56 to 98.3% ± 0.84 for several solid etoricoxib dispersions. Furthermore, the drug content in relation to the theoretical value ranged from 81.11% ± 1.41% to 98.73% ± 0.04 in a variety of freshly prepared etoricoxib solid dispersions. Table 4 summarizes the results.

### 3.2. Saturation Solubility

Pure etoricoxib showed a saturation solubility of 73.85 ± 2.19 μg/mL at pH 1.2 and 71.106 μg/mL ± 1.259 at pH6.8. The drug solubility of all samples, both physical mixtures and solid dispersions of etoricoxib in cyclodextrin derivatives, increased (Table 5).

### 3.3. FTIR Spectroscopy Analysis

The physicochemical interactions between etoricoxib and cyclodextrin-derivative carriers in their solid dispersions were investigated using FTIR spectroscopy.

**Figure 1:**
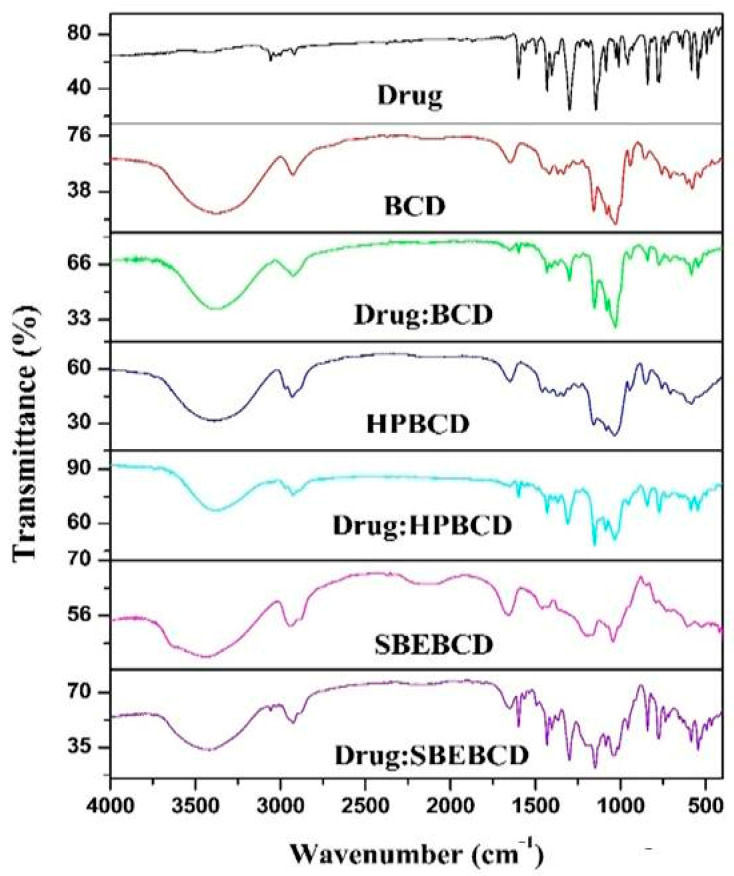
Fourier-transform–infrared (FTIR) spectra of pure etoricoxib, pure β-CD, pure HP β-CD, and pure SBE β-CD, and solid dispersions of etoricoxib at 1:1 weight ratio with β-CD, HP β-CD, and SBE β-CD.

Figure 1 shows the FTIR spectra of solid etoricoxib dispersions and pure etoricoxib. The characteristic peaks in the FTIR spectra of pure etoricoxib were observed at 1600.92 cm^−1^ (C=N stretching vibration); 1431.18, 1300.02 cm^−1^ (S=O stretching vibration); and 840.96, 771.53 cm^−1^ (C-Cl stretching vibration).

The FTIR spectrum of the solid dispersions of etoricoxib with cyclodextrin derivatives, such as β-CD, HP β-CD, and SBE β-CD, showed a shift and slight broadening of the -OH stretching at 3350.7 cm^−1^. With HP β-CD, a shifting and broadening of the S=0 stretching vibration to 1311.59 cm^−1^ was noticed.

### 3.4. DSC Analysis

**Figure 2:**
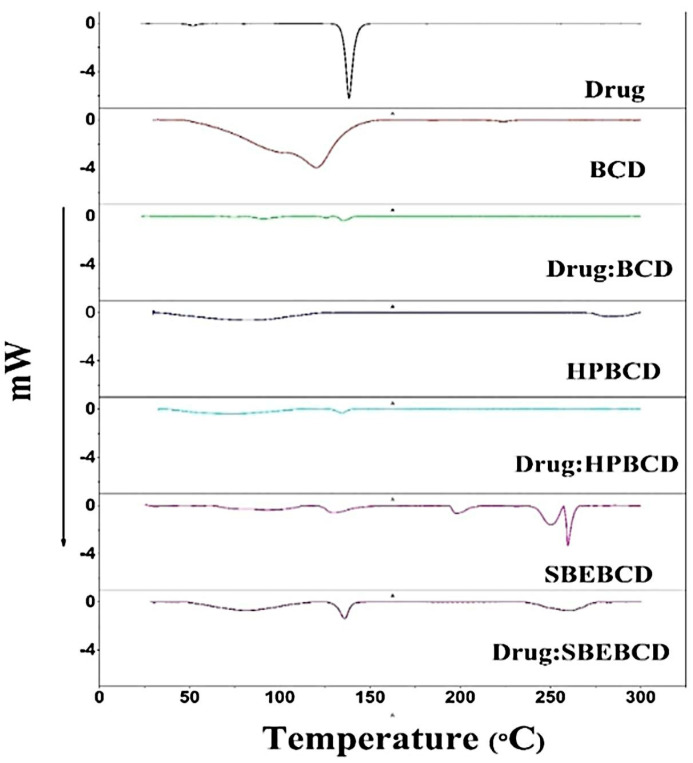
DSC thermogram of pure etoricoxib; pure β-CD; a etoricoxib: β-CD mixture; pure HP β-CD; etoricoxib: HP β-CD; pure SBE β-CD; and etoricoxib: SBE β-CD at a 1:1 weight ratio.

The melting pattern and crystalline properties of pure etoricoxib and its solid dispersions with β-CD, HP β-CD, and SBE β-CD were examined using DSC. Figure 2 shows DSC thermograms. A sharp endothermic peak at 139.88 °C was observed in the DSC thermogram of pure etoricoxib, which occurred due to drug melting. β-CD and HP β-CD broadened the drug endothermic peak compared to SBE β-CD. A decrease in peak intensity and a drop in enthalpy were also recorded from 62.6 J/g in the pure drug to 16.4 J/g in SBE β-CD, 3.93 J/g in β-CD, and 3.6 J/g in HP β-CD.

### 3.5. In Vitro Dissolution

**Figure 3:**
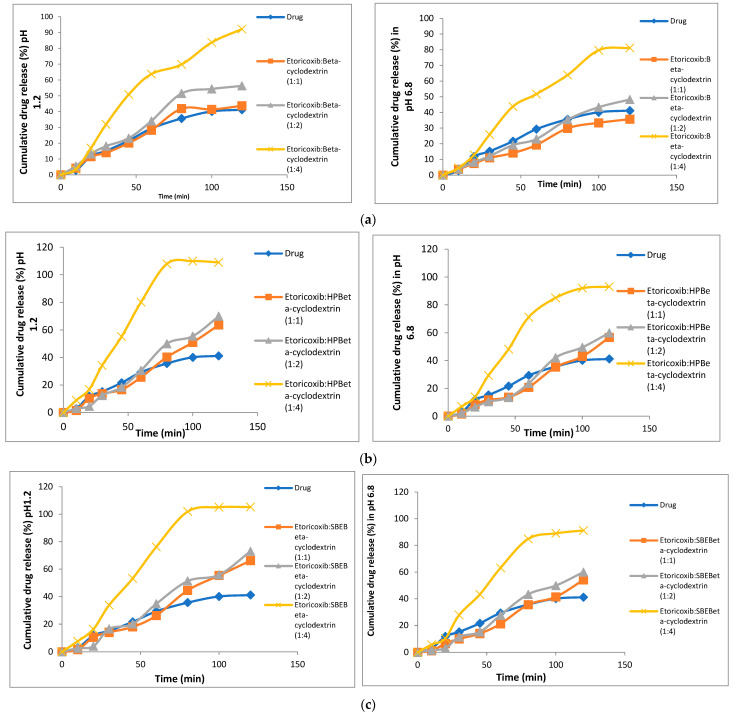
In vitro dissolution profiles of etoricoxib solid dispersions using β-CD (**a**), HP β-CD (**b**), and SBE β-CD (**c**) compared to pure etoricoxib (ET) in two different media, pH 1.2 and pH 6.8.

**Figure 4:**
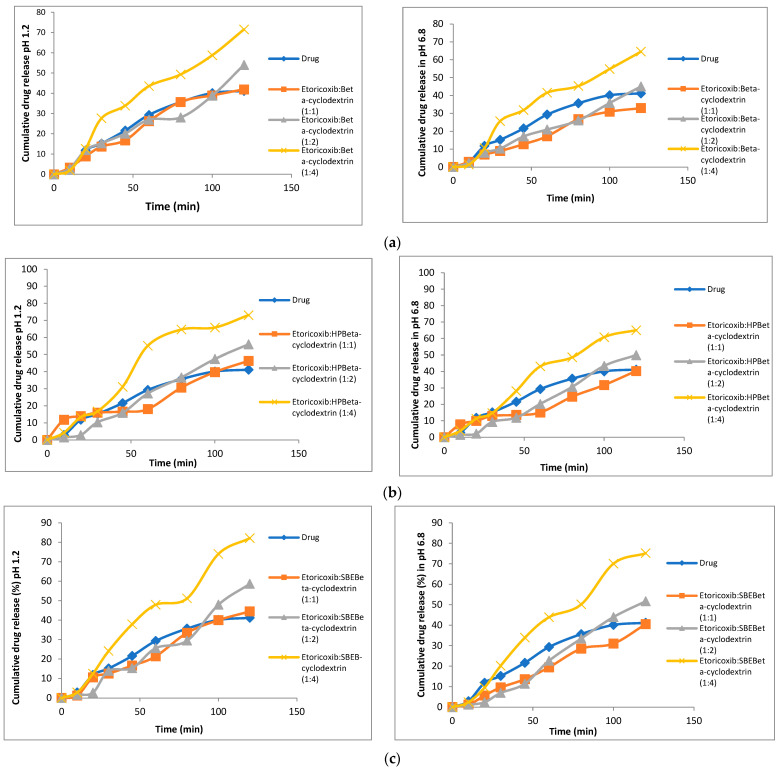
In vitro dissolution profiles of etoricoxib physical mixtures using β-CD (**a**), HP β-CD (**b**), and SBE β-CD (**c**) compared to etoricoxib in two different media, pH1.2 and pH6.8.

The drug (etoricoxib) in vitro dissolution profiles and those of different solid dispersions and their respective physical mixtures in phosphate buffer (pH = 6.8) and 0.1 N HCL (pH 1.2) are demonstrated in Figure 3 and Figure 4. All the physical mixtures and solid dispersion samples showed a significant (*p* = 0.048) increase in dissolution rate compared to pure etoricoxib. Figure 3 and Figure 4 demonstrate the dissolution profiles for etoricoxib solid dispersions and their physical mixtures.

### 3.6. Characterization of Fast-Dissolving Tablets of Etoricoxib

#### Evaluation of the Powder Blend for Tablet Formulations

The results of evaluation tests performed on solid dispersion powder blends are summed up in Table 6.

Based on the findings of powder blends’ flow properties, all of the suggested formulations were compressed to prepare the fast-dissolving tablets of etoricoxib. The prepared tablets were white in color and free from odor. All the tablet formulations succeeded in masking the taste of the drug. Physical parameters for the prepared tablet formulations are listed in Table 7.

The results of the in vitro disintegration and dissolution of etoricoxib at pH 1.2 and 6.8 are summarized in Table 8.

Table 9 sums up the rest of the parameters of the fast-dissolving tablet formulations.

**Figure 5:**
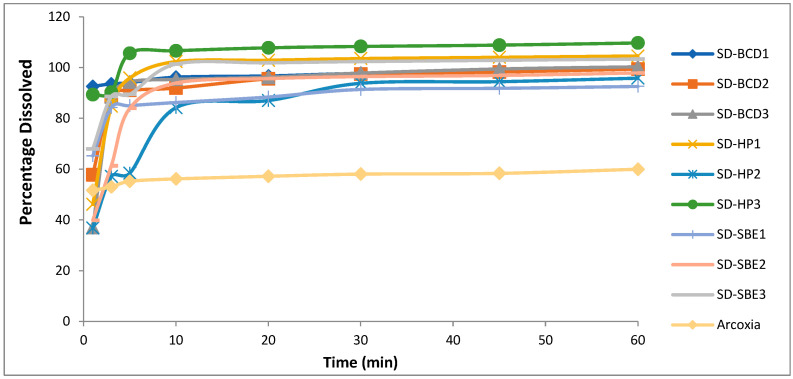
In vitro dissolution profiles of the fast-dissolving tablet formulations against Arcoxia^®^ in 0.1 N HCL (pH 1.2).

**Figure 6:**
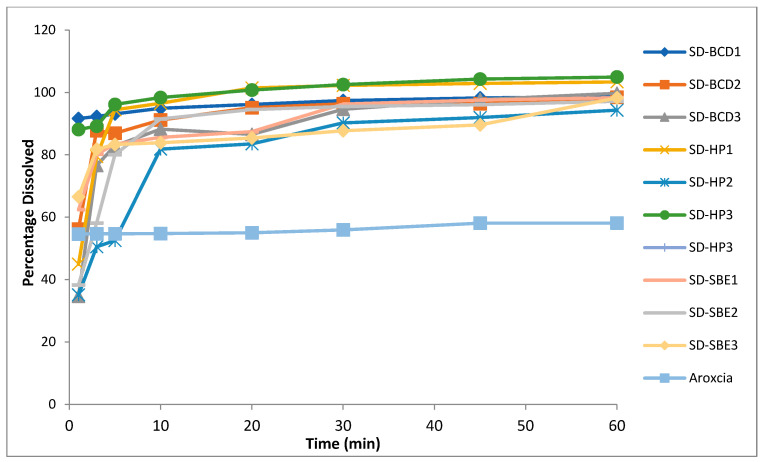
In vitro dissolution profiles of the fast-dissolving tablet formulations against Arcoxia^®^ inpPhosphate buffer (pH 6.8).

The release profiles of fast-dissolving etoricoxib tablets in 0.1 N HCL (pH 1.2) and in phosphate buffer (pH 6.8) compared to the marketed product Arxocia^®^ are demonstrated in Figure 5 and Figure 6, respectively. Dissolution profiles showed significantly higher dissolution rates (*p* = 0.045) for solid etoricoxib dispersions’ fast-dissolving tablets compared to the marketed product Arcoxia^®^ in both media.

**Figure 7:**
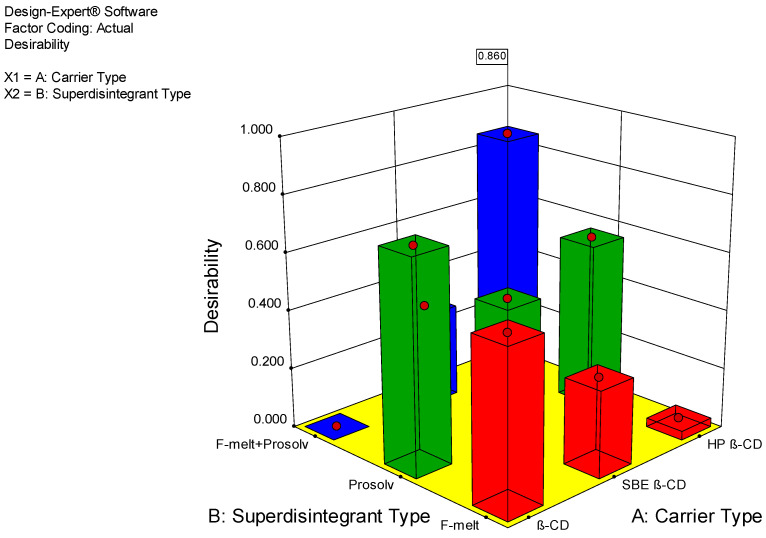
Desirability of fast-dissolving tablets according to Design Expert^®^.

Based on the results of the above parameters, which were taken as responses to the factorial design, the desirability of formulations was calculated and is demonstrated in Figure 7. As is clear from the figure, SD HP3 had the highest desirability (0.86).

### 3.7. In Vivo Study

**Figure 8:**
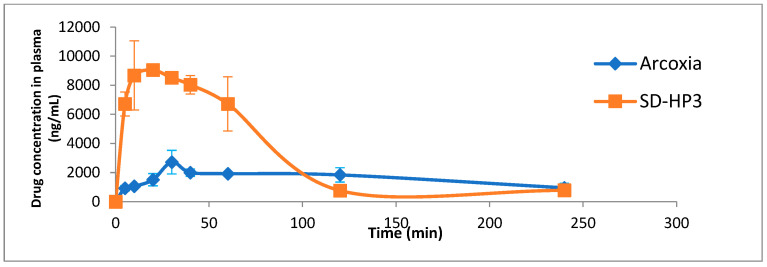
Plasma concentration vs. time profile after the administration of an oral dose of the selected formulation (SD-HP3) and the marketed product Arcoxia^®^ to rabbits, mean ± SD, n = 12.

Formulation SD-HP3 (Formulation #6), with the highest desirability, was used to evaluate the bioavailability of etoricoxib solid dispersion against the marketed product Arcoxia^®^. Table 10 summarizes the bioavailability parameters. Figure 8 shows the plasma concentration–time curves for both SD-HP3 and Arcoxia^®^.

Both Table 10 and Figure 8 demonstrated the superiority of the bioavailability of SD-HP3 over the marketed product. Following the administration of the SD-HP3 tablet and Arcoxia^®^, the results revealed that the extent of absorption was increased by around 1.4-fold in SD-HP3 compared to Arcoxia^®^. Furthermore, with SD-HP3, the maximum plasma concentration appeared rapidly with SD-HP3 (T_max_ = 13.3 ± 5.7 min) in comparison to the marketed tablet (T_max_ = 40 ± 17 min). These findings indicate that the absorption rate and bioavailability of SD-HP3 were significantly faster and greater than those of the marketed medication Arcoxia^®^.

## 4. Discussion

Because the solubility of etoricoxib is pH-dependent [36,37], the pH variation could therefore skew the results of the solubility measurement and in vitro drug-release study. To maintain a constant pH, phosphate buffers at pH 6.8 and pH 1.2 were employed as media to examine etoricoxib’s saturation solubility and dissolution pattern. The saturation solubility of etoricoxib solid dispersions was substantially higher than that of physical drug–carrier mixtures (*p* = 0.047). That was due to the water-soluble cyclodextrin derivatives acting as carriers, improving drug particle wetting and localized solubilization. Also, cyclodextrin possessed increased water solubility, as well as amorphizing, wetting, solubilizing, and complexing power [38]. The order of increasing saturation solubility by using cyclodextrin derivatives was found to be as follows: HP β-CD > SBE β-CD > β-CD. A low solubility associated with mixing the drug with β-CD was mostly due to the poor solubility of β-CD [38].

The solid dispersion formulation named SD-HP3 prepared using a solvent evaporation technique with a 1:4 ratio showed a maximum dissolution rate compared to the solid dispersion prepared with other weight ratios of 1:1 and 1:2. An inclusion complex was formed upon mixing the drug with the cyclodextrins at such a ratio by weight through non-covalent bonding, such as van der Waals interactions, hydrophobic effect, solvent re-organization, and hydrogen bonding [15,16]. That was due to the fact that such a ratio corresponded to a 1:1 molar ratio at which an inclusion complex formed [14]. β-CD derivatives possessed a lipophilic central cavity and a hydrophilic exterior [17] and increased the surface available for dissolution by reducing the interfacial tension between hydrophobic drug and dissolution media, leading to increased drug solubility, stability, and bioavailability. Also, they possessed powerful complexation and the ability to enhance water solubility [39]. At low drug: carrier weight ratios (1:1 and 1:2), the drug would be partially encapsulated, which accounts for improved solubility and dissolution.

FTIR analysis recorded peak shifts and broadening. These might point to the potential for intermolecular hydrogen bonding between etoricoxib’s S=O and -OH groups and the O-H groups of carriers during the development of solid dispersions.

The observed changes in endothermic peaks during the DSC analysis could be attributable to the development of amorphous etoricoxib in solid dispersion [4,40]. The peak intensity and enthalpy reduction accounted for a decrease in the degree of the crystalline property of the drug [41]. Accordingly, a high reduction in the crystalline character, evidenced by the highest drop in enthalpy, accounted for the superior solubility of solid dispersion prepared from drug: HP β-cyclodextrin at a 1:4 weight ratio.

The in vitro drug release was carried out for both physical mixtures and solid dispersions in two different medium pHs of 1.2 and 6.8. The dissolution rate was higher at pH 1.2 than pH 6.8, and this was due to the weakly basic nature of etoricoxib (presence of a nitrogen atom within its structure) with a high solubility at a pH < 3 [42]. This confirmed that etoricoxib had a pH-dependent solubility.

The order for the increasing dissolution rate in phosphate buffer (pH = 6.8) and 0.1 N HCL (pH 1.2) was HP β-CD > SBE β-CD > β-CD. This observation was well correlated with the results of saturation solubility.

This was attributed to the drug being dispersed down to the molecular level in the dissolution medium. Molecular dispersion of the drug provided an increased surface area, which resulted in an increased dissolution rate and therefore improved the bioavailability [43]. Also, etoricoxib changed from its crystalline state to an amorphous form, which required no energy to break the lattice characteristic of crystalline drugs. Thus, drug dissolution was enhanced [44]. The disappearance of the crystal lattice structure within the prepared etoricoxib-SD systems increased the drug wetting due to reduced agglomeration [45].

The formulation SD-HP3 (1:4 ratio drug: carrier and equal amounts of super disintegrants) demonstrated a shorter disintegration time and a higher dissolution rate when compared to other formulations. Dissolution studies concluded that 107.8% of the drug was dissolved at the end of 20 min. The disintegration time increased in the order of β-CD < SBE β-CD < HP β-CD< etoricoxib, when present alone or as a physical mixture and exhibited a very low aqueous solubility, poor dissolution rate, delayed release, and delayed onset of action. Upon complexation at a 1:4 ratio with HP β-CD, the release of the drug was markedly increased, which was evident from the values listed in the pre-formulation study results.

Complexation of the drug with HP β-CD and the use of an equal mixture of Prosolv^®^ and F-melt^®^ exhibited the most desirable results and a more favored response than using any of the super-disintegrants individually. This was due to the properties of both super-disintegrants. Prosolv^®^ is a co-processed excipient consisting of 60%–70% D-mannitol, 15%–30% microcrystalline cellulose, 4%–6% fructose, 4%–6% crospovidone, and 1.5%–2.5% colloidal silicon dioxide [46]. Prosolv^®^ exhibited better flowability and compatibility than plain microcrystalline cellulose [45,47]. In addition, being mannitol-based, it facilitated wetting and drug release [48]. F-melt^®^ was designed for manufacturing fast-dissolving tablets through simple mixing with active pharmaceutical ingredients and lubricants. It also possessed outstanding tableting properties and had the advantage of short disintegration times within 30 s. It was cost-effective, resulted in less sticking or capping, and had a pleasant mouthfeel. Hence, both disintegrants had a synergistic effect. The chemical composition and amorphous character of HP β-CD accounted for its extreme water solubility [49]. The acceptable taste of tablets is attributed to masking the taste of the drug through its shielding within the inclusion complex [50].

The in vivo animal study in rabbits revealed higher levels of etoricoxib from the optimum formulation in serum compared to Arcoxia^®^. Tablets prepared with HP β-CD provided a more rapid onset of pharmacological effects in comparison to the market formulation and the pure drug due to the formation of a stable amorphous solid dispersion with increased solubility and dispersibility, resulting in the enhancement of the drug absorption and bioavailability.

## 5. Conclusions

It was possible to prepare a solid etoricoxib dispersion using the solvent-evaporation method with enhanced solubility and dissolution rates. Cyclodextrin derivatives provided good opportunities for the development of stable amorphous solid dispersions. Etoricoxib/HP β-CD solid dispersion, prepared at a 1:4 drug:carrier ratio, had the optimal saturation solubility, disintegration time, drug release after one and ten minutes, and the highest dissolution efficiency. The superiority of these physical parameters was confirmed to have an impact on the bioavailability of the drug through the in vivo study results. The etoricoxib formulation as a solid dispersion resulted in a considerable increase in the AUC, reduction in the time to peak concentration, and an increase in T_max_ compared to the marketed product Arcoxia^®^. The enhanced solubility of cyclodextrin helped to improve the properties of etoricoxib. The potential outcomes of these studies suggest that the inclusion complex represents a valuable approach for developing a better oral dosage form than that currently available in the commercial market, which, if scaled up, could be promising for the development of formulations of other poorly water-soluble drugs from the standpoint of industry.

## Figures and Tables

**Table 1:** The 3^2^ full-factorial-design factors and levels for the formulation of etoricoxib solid dispersion directly compressible tablets.

Factors (Independent Variables)	Levels		
Type of cyclodextrin	β-CD	HP β-CD	SBE β-CD
Directly compressible vehicle	Prosolv^®^ ODT	Prosolv^®^ ODT and F-melt	F-melt

**Table 2:** Formulation composition of fast-dissolving etoricoxib tablets.

Formulation	Formulation	Etoricoxib mg	BCD mg	HPBCD mg	SBEBCD mg	Prosolv^®^ mg	Fmelt^®^ mg	Total Weight mg
F1	SD-BCD1	60	240			300		600
F2	SD-BCD2	60	240				300	600
F3	SD-BCD3	60	240			150	150	600
F4	SD-HP1	60		240		300		600
F5	SD-HP2	60		240			300	600
F6	SD-HP3	60		240		150	150	600
F7	SD-SBE1	60			240	300		600
F8	SD-SBE2	60			240		300	600
F9	SD-SBE3	60			240	150	150	600

**Table 3:** The factorial-design responses and their constraints.

Response (Independent Variable)	Constraint
Hardness	Maximize
Friability	Minimize
Disintegration time	Minimize
Dissolution after one minute in phosphate buffer (pH 1.2 and 6.8)	Maximize
Dissolution after ten minutes in phosphate buffer (pH 1.2 and 6.8)	Maximize
Dissolution Efficiency	Maximize

**Table 4:** Percentage yield and percentage drug content of etoricoxib solid dispersions.

Solid Dispersion Type	Ratio	Percentage Yield % ± S.D	Percentage Drug Content % ± S.D
Etoricoxib: β-CD	1:1	82.5 ± 0.6	81.11 ± 1.41
	1:2	87.5 ± 0.4	83.03 ± 0.04
	1:4	96.3 ± 0.7	90.48 ± 0.03
Etoricoxib: HP β-CD	1:1	91.6 ± 0.8	94.71 ± 0.14
	1:2	88.8 ± 0.1	96.21 ± 0.41
	1:4	97.0 ± 1.4	98.32 ± 0.02
Etoricoxib: SBE β-CD	1:1	91.6 ± 0.8	96.11 ± 0.01
	1:2	93.3 ± 0.1	97.72 ± 0.42
	1:4	98.3 ± 0.8	98.73 ± 0.04

**Table 5:** Saturation solubility of pure etoricoxib, its different solid dispersions, and its physical mixtures.

Mixture	Ratio	Saturation Solubility μg/mL			
		Physical Mixture pH 1.2	Physical Mixture pH 6.8	Solid Dispersion pH 1.2	Solid Dispersion pH 6
Etoricoxib: β-CD	1:1	78.62 ± 0.82	76.30 ± 0.85	82.47 ± 2.02	81.36 ± 1.29
	1:2	80.00 ± 1.56	78.31 ± 0.86	86.51 ± 3.52	85.14 ± 0.76
	1:4	82.47 ± 0.75	81.11 ± 1.12	90.27 ± 2.58	88.63 ± 3.08
Etoricoxib: HP β-CD	1:1	90.13 ± 0.09	88.33 ± 0.01	110.91 ± 1.41	107.60 ± 1.84
	1:2	92.61 ± 0.57	91.79 ± 0.55	116.28 ± 4.78	115.30 ± 2.12
	1:4	105.41 ± 5.50	101.54 ± 0.76	131.55 ± 3.32	129.10 ± 1.27
Etoricoxib: SBE β-CD	1:1	93.44 ± 1.05	93.00 ± 1.27	115.18 ± 1.95	112.98 ± 1.99
	1:2	96.19 ± 0.07	95.51 ± 0.55	126.74 ± 2.97	111.36 ± 1.92
	1:4	103.89 ± 2.90	100.40 ± 0.71	123.85 ± 1.06	110.20 ± 0.71
Pure Etoricoxib in 0.1 N HCL pH(1.2)	73.85 μg/mL ± 2.20				
Pure Etoricoxib in Phosphate buffer pH(6.8)	71.12 μg/mL ± 1.23				

**Table 6:** Evaluation of the physical parameters of powder blends for preparation of fast-dissolving tablets of etoricoxib.

Formulation	Bulk Density	Tapped Density	Carr’s Index	Hausner’s Ratio	Angle of Repose
SD-BCD1	0.46 ± 0.03	0.5 ± 0.0	8.0 ± 0.6	1.094 ± 0.042	22.0 ± 2.8
SD-BCD2	0.44 ± 0.03	0.48 ± 0.01	8.33 ± 0.47	1.07 ± 0.02	21.0 ± 0.0
SD-BCD3	0.46 ± 0.02	0.49 ± 0.06	6.12 ± 0.03	1.05 ± 0.04	23.0 ± 0.7
SD-HP1	0.42 ± 0.02	0.44 ± 0.01	4.45 ± 0.07	1.06 ± 0.01	19.0 ± 1.4
SD-HP2	0.46 ± 0.01	0.49 ± 0.01	6.12 ± 0.17	1.048 ± 0.049	20.0 ± 0.7
SD-HP3	0.48 ± 0.01	0.52 ± 0.03	7.69 ± 0.18	1.068 ± 0.049	20.0 ± 0.0
SD-SBE1	0.45 ± 0.03	0.49 ± 0.01	8.163 ± 0.231	1.086 ± 0.003	19.0 ± 1.4
SD-SBE2	0.41 ± 0.02	0.45 ± 0.028	8.88 ± 0.62	1.067 ± 0.005	21.0 ± 0.7
SD-SBE3	0.45 ± 0.01	0.490 ± 0.028	8.16 ± 0.08	1.080 ± 0.113	22.0 ± 0.7

**Table 7:** Physical parameters of fast-dissolving tablets of etoricoxib.

Formulation	Weight Variation	Hardness (Kg)	Drug Content (%)	Friability (% Weight Loss)
SD-BCD1	Passes	5.20 ± 0.28	98.1 ± 1.3	0.44 ± 0.03
SD-BCD2	Passes	4.25 ± 0.14	99.6 ± 0.9	0.425 ± 0.028
SD-BCD3	Passes	4.90 ± 0.28	98.3 ± 0.4	0.51 ± 0.01
SD-HP1	Passes	5.00 ± 0.42	98.9 ± 1.4	0.40 ± 0.01
SD-HP2	Passes	4.85 ± 0.28	100.3 ± 0.4	0.525 ± 0.028
SD-HP3	Passes	4.50 ± 0.14	98.7 ± 0.3	0.46 ± 0.01
SD-SBE1	Passes	5.40 ± 0.42	98.7 ± 0.7	0.380 ± 0.014
SD-SBE2	Passes	4.60 ± 0.28	99.1 ± 0.4	0.440 ± 0.028
SD-SBE3	Passes	4.60 ± 0.71	99.3 ± 1.8	0.470 ± 0.028

**Table 8:** In vitro disintegration and dissolution of fast-dissolving tablets of etoricoxib.

Formulation	Disintegration Time in 0.1 N HCL pH 1.2 (sec)	Disintegration Time in Phosphate Buffer pH 6.8 (sec)	Percentage Drug Dissolved after 1 min in 0.1 N HCL Buffer pH 1.2	Percentage Drug Dissolved after 1 min in Phosphate Buffer pH 6.8	Percentage Drug Dissolved after 10 min in 0.1 M HCL Buffer pH 1.2	Percentage Drug Dissolved after 10 min in Phosphate Buffer pH 6.8
SD-BCD1	37.0 ± 0.4	43.0 ± 1.4	92.6 ± 0.7	91.6 ± 1.3	96.3 ± 1.3	94.9 ± 0.7
SD-BCD2	45.0 ± 1.4	47.5 ± 1.4	57.8 ± 2.0	56.4 ± 1.6	92 ± 0.4	91.1 ± 0.3
SD-BCD3	45.0 ± 2.8	49.0 ± 4.2	37.0 ± 0.3	34.7 ± 3.1	95.4 ± 0.7	88.2± 1.8
SD-HP1	60.0 ± 4.2	52.0 ± 2.8	46.2 ± 3.7	44.9 ± 2.8	102.0 ± 2.0	96.5± 2.0
SD-HP2	80.0 ± 2.8	72.0 ± 1.4	36.8 ± 2.8	35.1 ± 1.4	84.3 ± 1.7	81.8± 0.4
SD-HP3	25.0 ± 0.0	30.0 ± 7.1	89.4 ± 4.0	88.1 ± 0.3	100.7 ± 0.4	98.3± 7.1
SD-SBE1	33.0 ± 1.4	42.0 ± 8.5	65.2 ± 4.8	62.4 ± 0.6	86.2 ± 1.8	85.6± 2.8
SD-SBE2	70.0 ± 7.1	66.0 ± 7.1	39.8 ± 0. 6	38.3 ± 4.0	93.9 ± 0.7	91.5± 1.0
SD-SBE3	52.0 ± 1.4	48.0 ± 8.5	67.9 ± 1.4	66.5 ± 0.4	101.0 ± 2.0	83.9± 3.5
Arcoxia^®^	120.0 ± 2.7	135.0 ± 1.5	51.7 ± 1.0	54.6 ± 2.6	56.2 ± 1.7	54.8± 1.3

**Table 9:** Taste masking, thickness, dissolution efficiency, and desirability of etoricoxib.

Formulation	Taste Masking	Thickness (mm)	Dissolution Efficiency %	Desirability
SD-BCD1	Accepted	4	93.936	0.734
SD-BCD2	Accepted	4	90.855	0.561
SD-BCD3	Accepted	4	80.370	Was excluded
SD-HP1	Accepted	4	96.487	0.573
SD-HP2	Accepted	4	81.979	0.031
SD-HP3	Accepted	4	98.344	0.860
SD-SBE1	Accepted	4	85.920	0.456
SD-SBE2	Accepted	4	91.917	0.298
SD-SBE3	Accepted	4	83.851	0.324

**Table 10:** The bioavailability parameters of etoricoxib and Arcoxia^®^.

Bioavailability Parameters	SD-HP3 ± S.D	Arcoxia^®^ ± S.D
T_max_ (minutes)	13.333 ± 5.773	40.0 ± 17.3
C_max_ (μg/mL)	9122.156 ± 225.508	2747.15 ± 767.48
AUC 0–240 (μg.min/mL)	541,863.4	375818.3
AUC 0-∞ μg.min/mL	854,200.1	665460.5
MRT (minutes)	62.070 ± 8.031	101.398 ± 7.106

S.D = standard deviation, n = 12.

## Data Availability

The data presented in this study are available in the article.
